# Celiac disease as a risk factor for pancreatitis: Evidence from multivariable Mendelian randomization and mediation analysis

**DOI:** 10.1097/MD.0000000000044445

**Published:** 2025-09-12

**Authors:** Hui Yang, Lu Ye, An Mao, Danyan Qian, Zhimei Hu

**Affiliations:** a Outpatient Department, The 903th Hospital of the Joint Logistics Support Force of the People's Liberation Army, Hangzhou, China; b Jiangxi Provincial People’s Hospital, The First Affiliated Hospital of Nanchang Medical College, Nanchang, China.

**Keywords:** celiac disease, genome-wide association study, mediation analysis, Mendelian randomization, pancreatitis

## Abstract

Previous studies have suggested a potential link between celiac disease (CeD) and an increased risk of pancreatitis. However, the causal relationship and underlying mechanisms remain unclear. This study utilizes multivariable Mendelian randomization (MVMR) and mediation analysis to explore the causal link and underlying pathways between CeD and pancreatitis. Inverse variance weighting (IVW) was employed as the primary analysis method in MR analysis. To account for the potential confounding effects of cholelithiasis and triglycerides (TG), MVMR was further conducted. Furthermore, the mediating role of Sjögren syndrome (SS) in the relationship between CeD and pancreatitis was examined. Finally, multiple sensitivity analyses were utilized to assess the robustness of the results. Meta-analysis of the IVW results indicated that CeD is a potential risk factor for acute pancreatitis (AP) (pooled OR_IVW_ = 1.05, 95% CI = 1.03–1.08, *P* < .001) and chronic pancreatitis (CP) (pooled OR_IVW_ = 1.07, 95% CI = 1.04–1.10, *P* < .001). The MVMR result suggested the causal effects of CeD on AP (OR_IVW_ = 1.05, 95% CI = 1.03–1.08, *P* < .001) and CP (OR_IVW_ = 1.07, 95% CI = 1.03–1.10, *P* < .001) remain significant even after adjusting for cholelithiasis and TG. Further mediation analysis revealed that SS plays a mediating role in the causal effect of CeD on AP (OR = 1.01, 95% CI = 1.00–1.02, *P* = .033). The final sensitivity analysis showed no significant heterogeneity or horizontal pleiotropy. This study strongly supports a causal link between CeD and pancreatitis, both AP and CP, with SS potentially mediating the CeD-AP relationship.

## 1. Introduction

Celiac disease (CeD) is a chronic autoimmune condition that is initiated by the ingestion of gluten by genetically susceptible individuals, particularly children and young adults.^[1,2]^ It impacts about 1% of the global population,^[3,4]^ with higher prevalence in females.^[5]^ The pathogenesis of CeD is complex, involving genetic susceptibility, abnormal immune system response, and environmental factors.^[6–11]^ CeD is typically characterized by malabsorption of various nutrients, villous atrophy in the small intestine, and symptom improvement following the gluten-free diet, which exhibits considerable variability in clinical manifestations and could affect multiple organ systems. Common symptoms include abdominal bloating, weight loss, chronic diarrhea and constipation.^[12]^ Some patients may present with atypical forms of the disease or remain asymptomatic.^[13,14]^

Compared to intestinal manifestations, the spectrum of extraintestinal involvement in CeD may be more diverse and unpredictable.^[15]^ Recent clinical studies suggest that CeD may pose an increased risk of pancreatitis. A large cross-sectional study employing clinical databases revealed that the likelihood of pancreatitis in individuals with CeD is more than twice as high as in those without CeD.^[16]^ Similarly, another cohort study also demonstrated that the risk of pancreatitis in individuals with CeD is nearly 3 times higher than control group.^[17]^

However, it is noteworthy that observational studies are prone to various formative factors such as environmental, metabolic, and medication influences, making it difficult to infer true causal relationships. Furthermore, current epidemiological studies could not uncover the underlying mechanisms behind this association. Therefore, we undertook Mendelian randomization (MR) to further investigate the impact of CeD on pancreatitis. MR, as a novel epidemiological method, utilizes genetic variation as the principal variable (instrumental variables (IVs)) to study causal relationships between risk factors and disease outcomes.^[18]^ Given that the allocation of genetic variation at the time of conception is random, MR study is less affected by environmental factors and other confounding variables.^[19]^ Compared to observational studies, MR provides stronger causal evidence and helps reveal the true relationship. Recently, MR has become widely employed, particularly in assessing genetic link between complex diseases, offering a valuable tool for epidemiological research.^[20]^

In this MR study, both univariable MR (UVMR) and multivariable MR (MVMR) analyses were conducted to assess the association between CeD and 4 subtypes of pancreatitis. As CeD is often found to coexist with other autoimmune diseases,^[21,22]^ such as autoimmune thyroiditis,^[23]^ systemic lupus erythematosus,^[24]^ juvenile idiopathic arthritis,^[25]^ type 1 diabetes,^[26]^ and particularly Sjögren syndrome (SS),^[27]^ a predominantly exocrine autoimmune disorder, has been reported to be a candidate marker for pancreatitis. Therefore, we further evaluated whether SS may mediate the increased risk of pancreatitis associated with CeD. Overall, this MR study not only examined the genetic link between CeD and pancreatitis but also explored possible underlying mechanisms contributing to this relationship.

## 2. Materials and methods

### 2.1. Study design

**Figure 1.**
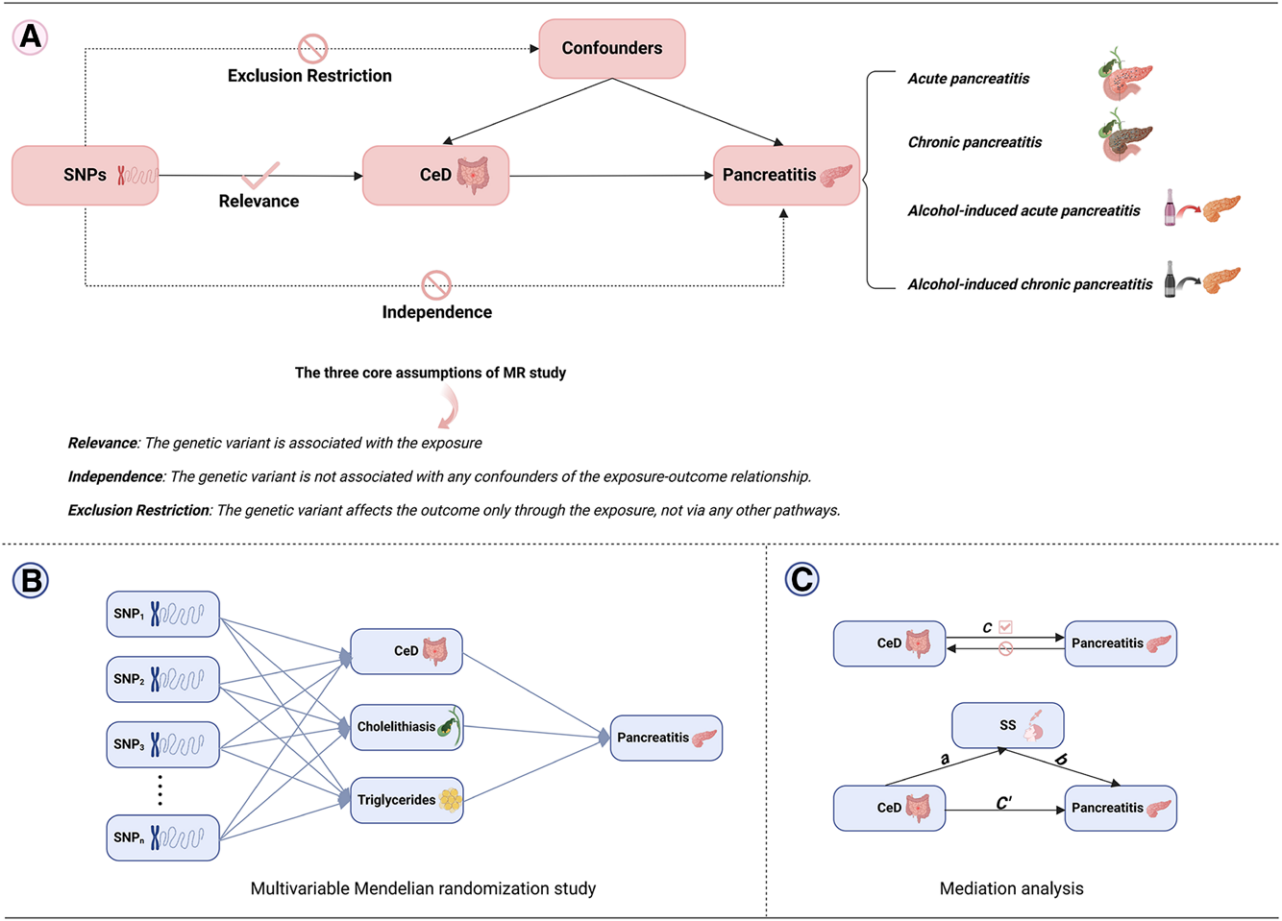
Diagram of MR methods utilized in this study. (A) Diagram of two-sample MR. (B) Diagram of multivariable MR. (C) Diagram of mediation analysis. CeD = celiac disease, MR = Mendelian randomization, SNPs = single nucleotide polymorphisms, SS = Sjögren syndrome.

Firstly, based on the 3 core assumptions of MR,^[28]^ UVMR was employed to investigate the genetic causal effects of CeD on 4 subtypes of pancreatitis including acute pancreatitis (AP), chronic pancreatitis (CP), alcohol-induced acute pancreatitis (AIAP), and alcohol-induced chronic pancreatitis (AICP), in both discovery and replication cohorts (Fig. 1A). Subsequently, a meta-analysis was conducted on the inverse variance weighting (IVW) results from the 2 cohorts. Furthermore, cholelithiasis and triglycerides (TG) were included in MVMR to analyze the independent effect of CeD on pancreatitis (Fig. 1B). Finally, mediation analysis was used to explore the mediating effect and proportion of SS in the causal relationship between CeD and pancreatitis (Fig. 1C).

### 2.2. Data sources

The genome-wide association studies (GWAS) data on CeD for both the discovery and replication cohorts were obtained from the IEU database,^[29]^ which is an online repository for publicly accessible GWAS summary data (https://gwas.mrcieu.ac.uk/), with IDs of “ebi-a-GCST005523” (sample size = 23,649) and “ieu-a-276” (sample size = 15,283), respectively. The GWAS summary data for the 4 subtypes of pancreatitis were derived from the Finnish Biobank (https://www.finngen.fi/en),^[30]^ which include extensive collections of DNA, blood, and tissue samples, as well as linked health records from a wide range of Finnish populations supporting various types of medical and genetic research. Cholelithiasis GWAS data were obtained from a whole-genome regression analysis of the UK Biobank dataset conducted by Mbatchou et al in 2021 (sample size = 404,405).^[31]^ Furthermore, the global lipids genetics consortium provided the GWAS data for TG (sample size = 177,861).^[32]^ Finally, we additionally acquired the GWAS data SS from the IEU database, with IDs of “ebi-a-GCST90018920” (sample size = 484,013). A summary of all GWAS summary data utilized in this MR study is provided in Table 1.

**Table 1:** Detailed GWAS information utilized in this MR study.

Trait	ID (source)	Year	Sample size	Population
CeD (discovery cohort)	ebi-a-GCST005523 (IEU)	2011	23,649	European
CeD (replication cohort)	ieu-a-276 (IEU)	2010	15,283	European
AP	finngen_R10_K11_ACUTPANC (FinnGen)	2023	368,428	European
CP	finngen_R10_K11_CHRONPANC (FinnGen)	2023	365,516	European
AIAP	finngen_R10_ALCOPANCACU (FinnGen)	2023	412,181	European
AICP	finngen_R10_ALCOPANCCHRON (FinnGen)	2023	412,181	European
Cholelithiasis	ebi-a-GCST90013889 (IEU)	2021	404,405	European
TG	ieu-a-302 (IEU)	2013	177,861	Mixed*
SS	ebi-a-GCST90018920 (IEU)	2021	484,013	European

AIAP = alcohol-induced acute pancreatitis, AICP = alcohol-induced chronic pancreatitis, AP = acute pancreatitis, CeD = celiac disease, CP = chronic pancreatitis, GWAS = genome-wide association study, MR = Mendelian randomization, SS = Sjögren syndrome, TG = triglycerides.

* Europeans account for about 90%.

### 2.3. Selection of IVs

**Figure 2.**
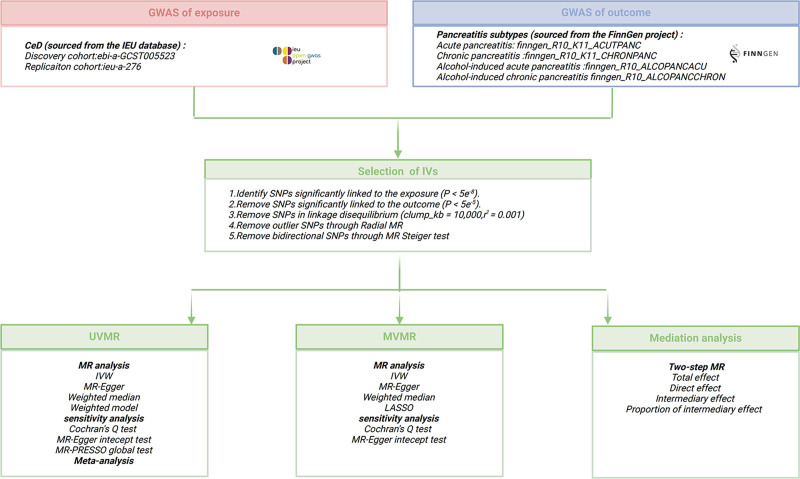
Flowchart of this Mendelian randomization. CeD = celiac disease, GWAS = genome-wide association study, IVW = inverse variance weighted, MR-PRESSO = Mendelian randomization pleiotropy residual sum and outlier, MVMR = multivariable Mendelian randomization, SNPs = single nucleotide polymorphisms, UVMR = univariable Mendelian randomization.

Initially, to ensure that single nucleotide polymorphisms (SNPs) could effectively serve as IVs for CeD, SNPs not significantly associated with CeD were excluded (*P* > 5 × 10^−8^). Secondly, based on the independence assumption, only SNPs not in linkage disequilibrium were retained (kb = 10,000, *r*^2^ = 0.001). Additionally, to avoid the potential adverse effects of weak IVs on MR analysis, the *F*-statistic for each SNP was calculated (*F*=β^2^/se^2^),^[33]^ and SNPs with an *F*-value < 10 were filtered out. Furthermore, to ensure the homogeneity of SNPs, Radial MR was utilized to exclude SNPs with significant heterogeneity^[34]^ (Fig. S1, Supplemental Digital Content, https://links.lww.com/MD/P922, Table S1, Supplemental Digital Content, https://links.lww.com/MD/P923). Finally, the MR Steiger directionality test was applied to eliminate SNPs with reverse causality effects^[35]^ (Fig. 2). The detailed information on the SNPs used as genetic proxies for CeD is summarized in Table S2, Supplemental Digital Content, https://links.lww.com/MD/P923.

### 2.4. MR analysis and statistical methods

When horizontal pleiotropy is absent in two-sample MR, IVW could provide relatively stable and accurate causal estimates by combining the causal effect estimates of each SNP using meta-analysis methods. Therefore, the IVW method is commonly used as the primary statistical method in MR analysis. However, ineffective IVs and pleiotropic effects are not accounted for by IVW. Thus, MR-Egger, weighted median, and weighted mode – 3 supplementary methods – were employed, with each method based on distinct assumptions regarding horizontal pleiotropy. Meta-analysis was adopted to assess the pooled effects of the IVW results from both the discovery and replication cohorts. When the *P*-value of Cochran *Q* test was <0.1 or *I*² > 50%, heterogeneity was considered present, and a random-effects model was applied. Otherwise, a fixed-effects model was used. As an extension of two-sample Mendelian randomization, MVMR estimates the causal impact of various risk factors on outcomes by including all exposures in the same model. To assess the direct impact of CeD on pancreatitis risk independent of cholelithiasis and TG, significant SNPs related to cholelithiasis and TG were extracted in our MVMR analysis. In addition to the aforementioned MR analysis methods, least absolute shrinkage and selection operator regression was additionally applied. Furthermore, we employed a two-step approach to analyze the mediating role of SS in the relationship between CeD and increased risk of pancreatitis. Sensitivity analysis included heterogeneity analysis and horizontal pleiotropy test. Cochran *Q* statistic was used to assess heterogeneity between SNP effect estimates. The MR pleiotropy residual sum and outlier (MR-PRESSO) global test and MR-Egger regression were employed to detect the presence of horizontal pleiotropy (Fig. 2). Additionally, leave-one-out analysis was performed to evaluate the influence of individual SNPs on the MR analysis results. All statistical analyses were conducted using R software (version 4.2.3). Considering that this MR study involves multiple comparisons, Bonferroni correction of *P*-values was applied to reduce the risk of false positives.^[36]^ *P*-values <.006 (.050/8) are considered statistically significant, while *P*-values between .006 and .050 are considered suggestive causal effect.

## 3. Result

### 3.1. SNPs as IVs in this MR analysis

Following the rigorous screening process described above, there were 37, 35, 38, and 33 SNPs serving as genetic proxies for CeD to analyze the causal effects of CeD on the 4 subtypes of pancreatitis in the discovery cohort. In the replication cohort, there were 12, 11, 12, and 12 SNPs acting as genetic proxies for CeD to investigate the causal effects of CeD on the 4 subtypes of pancreatitis. The *F*-values for all SNPs were >10 (Table S2, Supplemental Digital Content, https://links.lww.com/MD/P923).

### 3.2. The causal effects of CeD on pancreatitis subtypes in UVMR

**Figure 3.**
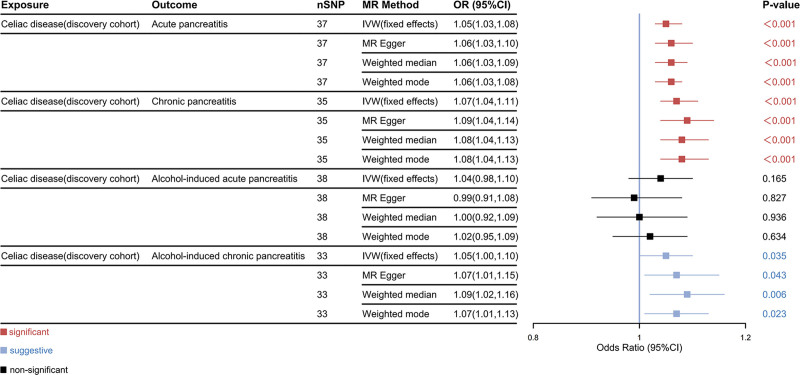
Causal effects of celiac disease on subtypes of pancreatitis in the discovery cohort. 95% CI = 95% confidence interval, MR = Mendelian randomization, nSNP = number of SNPs, OR = odds ratio, SNPs = single nucleotide polymorphisms.

**Figure 4.**
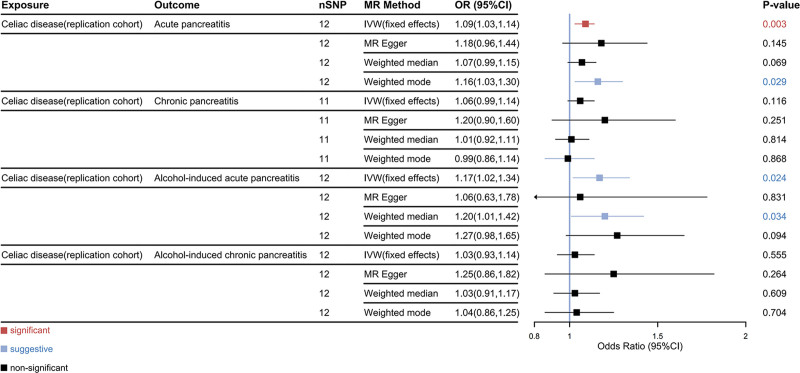
Causal effects of celiac disease on subtypes of pancreatitis in the replication cohort. 95% CI = 95% confidence interval, MR = Mendelian randomization, nSNP = number of SNPs, OR = odds ratio, SNPs = single nucleotide polymorphisms.

**Figure 5.**
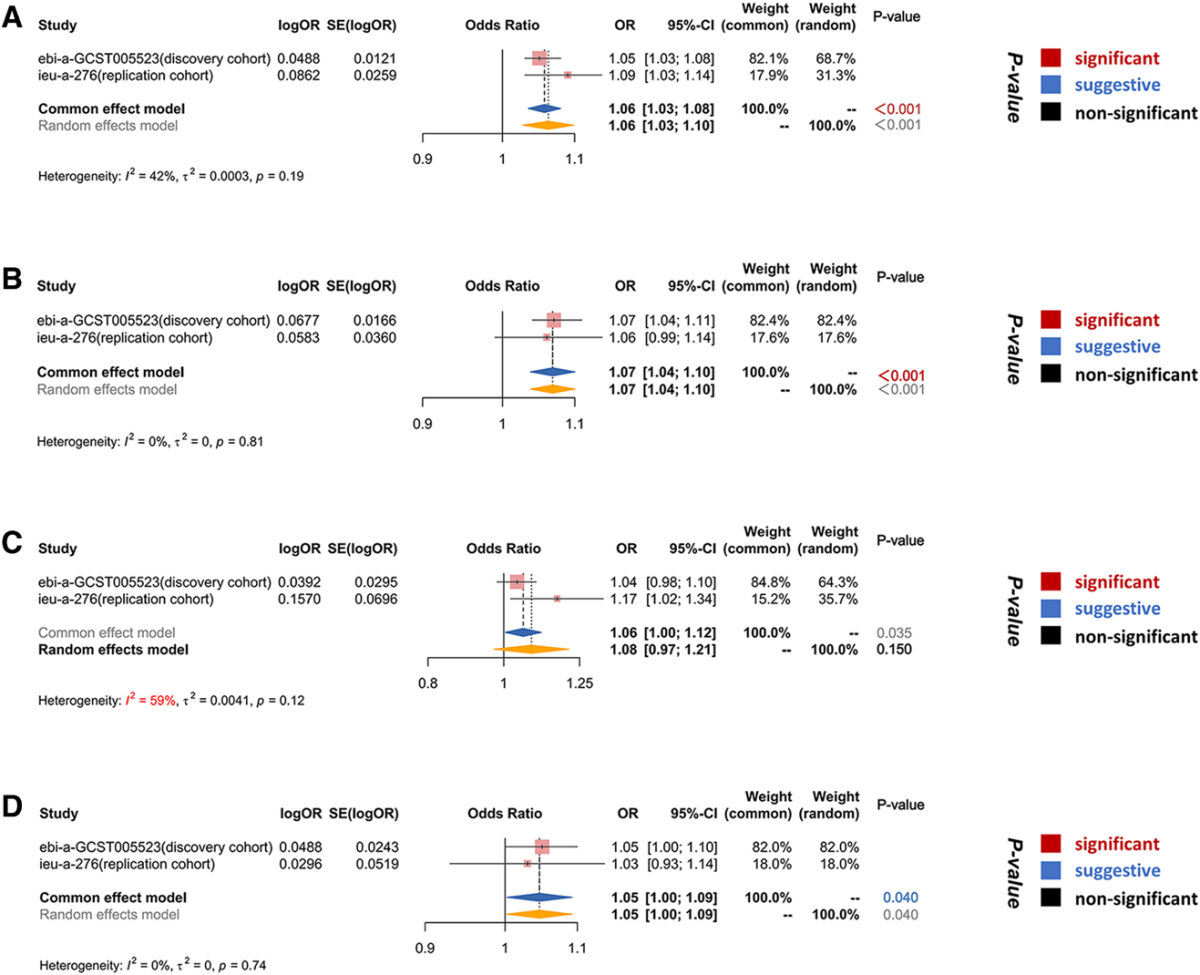
Meta-analysis of IVW results from the discovery and validation cohorts. 95% CI = 95% confidence interval, OR = odds ratio.

In the discovery cohort, MVMR results indicated that CeD has a significant causal effect on AP (odds ratio (OR)_IVW_ = 1.05, 95% confidence interval (CI): 1.03–1.08, *P* < .001) and CP (OR _IVW_ = 1.07, 95% CI = 1.04–1.11, *P* < .001), and a suggestive causal effect on AICP (OR _IVW_ = 1.05, 95% CI = 1.00–1.10, *P* = .035). However, there was not enough evidence to show that CeD increases the risk of AIAP (OR _IVW_ = 1.04, 95% CI = 0.98–1.10, *P* = .165) (Fig. 3). In the replication cohort, the IVW results still indicated that CeD significantly increases the risk of AP (OR_IVW_ = 1.09, 95% CI = 1.03–1.14, *P* = .003) and shows suggestive causal effect on AIAP (OR_IVW_ = 1.04, 95% CI = 0.98–1.10, *P* = .165), while no significant association with CP (OR_IVW_ = 1.17, 95% CI = 1.02–1.34, *P* = .024) or AICP (OR_IVW_ = 1.03, 95% CI = 0.93–1.14, *P* = .555) was found (Fig. 4). To ensure the reliability of the results, a meta-analysis was conducted to obtain the combined effect, which revealed that CeD is likely a risk factor for both AP (pooled OR_IVW_ = 1.05, 95% CI = 1.03–1.08, *P* < .001) and CP (pooled OR_IVW_ = 1.07, 95% CI = 1.04–1.10, *P* < .001). Additionally, there is suggestive evidence supporting a causal association between CeD and AICP (pooled OR_IVW_ = 1.05, 95% CI = 1.00–1.09, *P* = .04). However, no significant causal effect of CeD on AIAP was found (pooled OR_IVW_ = 1.08, 95% CI = 0.97–1.21, *P* = .150) (Fig. 5).

### 3.3. The independent causal effects of CeD on AP and CP in MVMR

**Figure 6.**
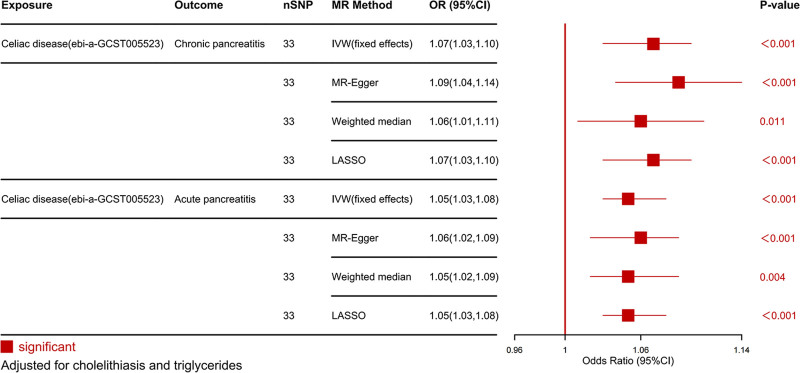
Causal effects of celiac disease on subtypes of pancreatitis in MVMR. 95% CI = 95% confidence interval, MR = Mendelian randomization, nSNP = number of SNPs, OR = odds ratio, SNPs = single nucleotide polymorphisms.

Further MVMR analysis was conducted on the significant causal relationships identified by UVMR. MVMR analysis showed that even after adjusting for cholelithiasis and TG, the causal effect of CeD on AP (OR_IVW_ = 1.05, 95% CI = 1.03–1.08, *P* < .001) and CP (OR_IVW_ = 1.07, 95% CI = 1.03–1.10, *P* < .001) remained significant (Fig. 6).

### 3.4. Mediation analysis of SS in the causal effects of CeD on AP and CP

**Figure 7.**
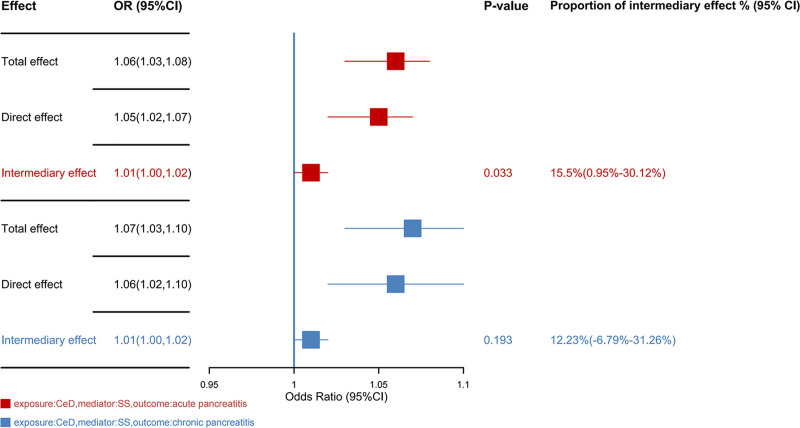
The result of mediation analysis. 95% CI = 95% confidence interval, OR = odds ratio, SNPs = single nucleotide polymorphisms.

Mediation analysis indicates that CeD may contribute to an elevated risk of AP by increasing the likelihood of developing SS (OR = 1.01, 95% CI = 1.00–1.02, *P* = .033) (Fig. 7). The proportion of intermediary effect is approximately 15.5% (95% CI = 0.95–30.12%). However, current evidence did not support that SS could act as a potential mediator in the causal relationship between CeD and CP (OR = 1.01, 95% CI = 1.00–1.02, *P* = .193) (Fig. 7).

### 3.5. Results of sensitivity analysis

Cochran *Q* test did not reveal any significant heterogeneity in our MR analysis (all *P* > .050). Additionally, leave-one-out analysis showed that our MR results were minimally influenced by any single SNP (Fig. S2, Supplemental Digital Content, https://links.lww.com/MD/P922). Sensitivity analyses, including the MR-Egger intercept and MR-PRESSO global tests, suggested no significant horizontal pleiotropy in all MR analyses (all *P* > .050) (Table 2).

**Table 2:** The results of heterogeneity analysis in UVMR and MVMR.

Outcome	Cochran *Q* test		Egger intercept test	MR-PRESSO
	*P*-value (MR–Egger)	*P*-value (IVW)	*P*-value	*P*-value of global test
UVMR				
Discovery cohort				
AP	.930	.937	.505	.935
CP	.593	.614	.465	.630
AIAP	.756	.698	.141	.698
AICP	.957	.955	.354	.936
Replication cohort				
AP	.718	.735	.432	.742
CP	.768	.776	.411	.760
AIAP	.889	.922	.706	.922
AICP	.763	.738	.312	.745
MVMR				
AP	.657	.702	.808	NA
CP	.191	.158	.174	NA

AIAP = alcohol-induced acute pancreatitis, AICP = alcohol-induced chronic pancreatitis, AP = acute pancreatitis, IVW = inverse variance weighted, MR-PRESSO = Mendelian randomization pleiotropy residual sum and outlier, MVMR = multivariable Mendelian randomization, NA = not available, UVMR = univariable Mendelian randomization.

## 4. Discussion

CeD is a chronic autoimmune lesion caused by an aberrant immune response to gluten in the small intestine,^[37]^ primarily affecting individuals with genetic susceptibility, particularly those with HLA-DQ8 and HLA-DQ2 genotypes,^[38]^ However, it is worth noting that, according to the latest European Society for Pediatric Gastroenterology, Hepatology, and Nutrition (ESPGHAN) guidelines, HLA genotyping is no longer considered essential for the diagnosis of CeD and should not be routinely used in the initial diagnostic workup.^[39]^ Although the classic symptoms of CeD are mainly gastrointestinal, such as chronic diarrhea and abdominal bloating, its extraintestinal manifestations are often more complex and easily overlooked. Recent years have seen increased attention to the extraintestinal manifestations of CeD,^[40]^ including skin rashes, arthritis, and osteoporosis. Pancreatitis, as another extraintestinal manifestation of CeD,^[41]^ is relatively under-researched. Our analyses using UVMR and MVMR methods indicate that CeD is a potential risk factor for AP and CP.

Our findings provide some support for previous clinical observational studies. A large-scale retrospective study including over 100,000 individuals with CeD reveals that these patients face more than double the risk of pancreatitis compared to those without the condition.^[42]^ Additionally, the study suggests that the risk of AP is relatively higher than that of CP. Another cohort study based on Swedish national registers data shows that CeD is associated with a significantly elevated risk of developing CP after diagnosis.^[43]^ Although our UVMR indicated causal effects of CeD on both AP and CP, the effect sizes were quite similar. Moreover, MVMR results demonstrated that the causal effect of CeD on pancreatitis was minimally influenced by cholelithiasis and TG.

There are multiple pathogenic mechanisms that can explain why CeD patients have an increased risk of pancreatitis. Malnutrition has a particularly significant impact on the pancreas.^[44]^ The pancreas is an organ highly dependent on nutritional supply, and inadequate nutritional intake can directly affect its cellular function, leading to secretion dysfunction and tissue atrophy.^[45]^ This not only makes the pancreas more susceptible to damage but could also lead to the development of pancreatitis. Papillary stenosis is another important factor. The chronic inflammatory response in CeD patients can cause stenosis of the duodenal papilla, which obstructs the normal flow of pancreatic juice, leading to increased pressure within the pancreas and raising the risk of pancreatitis.^[46]^ Furthermore, immune mechanisms also play a crucial role in CeD patients.^[47]^ Autoimmune pancreatitis is a disease caused by the immune system attacking the pancreas’s own tissue, and this is not uncommon among CeD patients. Previous research has suggested a possible link between CeD and SS,^[48]^ which is characterized by the involvement of exocrine glands, including the pancreas.^[49]^ Given this context, it is worth exploring whether CeD increases the risk of SS, thereby leading to pancreatitis. Therefore, we validated this hypothesis through mediation analysis. The mediation analysis revealed that approximately 15.5% of the causal effect of CeD on AP is mediated by SS. However, this mediating effect was not observed in CP. In conclusion, this finding highlights the importance of screening for other autoimmune diseases in patients with CeD. In those with concurrent pancreatitis, special attention should be given to SS, and an autoimmune antibody profile may help identify this potential risk.

Our study possesses several strengths. Firstly, subgroup analyses were conducted for 4 different subtypes of pancreatitis. Additionally, the potential influences of cholelithiasis and TG on pancreatitis were excluded using the MVMR approach. Furthermore, the intermediary role of SS in the pathway from CeD to pancreatitis was established through mediation analysis, which not only validates the associations previously identified by traditional observational studies but also reveals the potential underlying mechanisms. Additionally, meta-analysis was performed on the results of large-scale GWAS data from different sources, which has further strengthened the robustness of our interpretations. Finally, multiple sensitivity analyses were conducted, all of which showed no potential heterogeneity or horizontal pleiotropy in our study.

However, our MR study inevitably faces several limitations. Our study primarily involves European populations, and genetic variations across different populations may affect the estimation of causal relationships. Genetic heterogeneity could lead to inconsistencies in results among different populations, impacting the generalizability of the results. Future research could further validate whether our conclusions are applicable to other populations through cross-ethnic MR analyses. Furthermore, in addition to cholelithiasis and TG, other unobserved confounding factors should also be considered. Although MR aims to reduce confounding, unobserved factors or undetected interactions may still influence the results, limiting the accuracy of causal inferences. It is important to acknowledge that the precision of phenotype measurements, including disease diagnoses or biomarkers, could significantly affect MR results. Variability in phenotype assessment can introduce noise and reduce the ability to detect true causal relationships. Additionally, MR analysis typically adopts a linear relationship linking exposure and to outcome. However, relationships in the real world may be non-linear or complex, which could lead to misinterpretation of causal effects. Finally, although we identified that SS may act as a mediator linking CeD and AP, the underlying molecular mechanisms remain unclear. Further research is required to elucidate whether cytokines, gut microbiota, or metabolites are involved in this process.

## 5. Conclusion

Our MVMR analysis indicates that CeD could be a potential risk factor for both AP and CP. Further mediation analysis indicates that SS may act as a mediator in the increased risk of AP associated with CeD, thereby reinforcing the necessity of screening for SS in patients with pancreatitis. However, the current evidence requires further validation through multicenter clinical studies and molecular biology research.

## Acknowledgments

We want to acknowledge the participants and investigators of all the GWAS used in this study.

## Author contributions

**Conceptualization:** Zhimei Hu.

**Investigation:** Hui Yang, Lu Ye.

**Methodology:** Hui Yang, Lu Ye.

**Writing – original draft:** Hui Yang, Lu Ye, Danyan Qian.

**Writing – review & editing:** An Mao.

## Supplementary Material



## References

[1] Sahin Y. Celiac disease in children: a review of the literature. World J Clin Pediatr. 2021;10:53–71. 34316439 10.5409/wjcp.v10.i4.53 PMC8290992

[2] Lindfors K Ciacci C Kurppa K. Coeliac disease. Nat Rev Dis Primers. 2019;5:3. 30631077 10.1038/s41572-018-0054-z

[3] Rubin JE Crowe SE Celiac D Lebwohl B Rubio-Tapia A. Epidemiology, presentation, and diagnosis of celiac disease. Gastroenterology. 2021;160:63–75. 32950520 10.1053/j.gastro.2020.06.098

[4] Rubin JE Crowe SE. Celiac disease. Ann Intern Med. 2020;172:ITC1–16. 31905394 10.7326/AITC202001070

[5] Fasano A Catassi C. Celiac disease. N Engl J Med. 2012;367:2419–26. 23252527 10.1056/NEJMcp1113994

[6] Dieli-Crimi R Cénit MC Nunez C. The genetics of celiac disease: A comprehensive review of clinical implications. J Autoimmun. 2015;64:26–41. 26194613 10.1016/j.jaut.2015.07.003

[7] Abadie V Han AS Jabri B Sollid LM. New insights on genes, gluten, and immunopathogenesis of celiac disease. Gastroenterology. 2024;167:4–22. 38670280 10.1053/j.gastro.2024.03.042 PMC11283582

[8] Gianfrani C Auricchio S Troncone R. Adaptive and innate immune responses in celiac disease. Immunol Lett. 2005;99:141–5. 15876458 10.1016/j.imlet.2005.02.017

[9] D’Avino P Serena G Kenyon V Fasano A. An updated overview on celiac disease: from immuno-pathogenesis and immuno-genetics to therapeutic implications. Expert Rev Clin Immunol. 2021;17:269–84. 33472447 10.1080/1744666X.2021.1880320

[10] Serena G Lima R Fasano A. Genetic and environmental contributors for celiac disease. Curr Allergy Asthma Rep. 2019;19:1–10. 31321608 10.1007/s11882-019-0871-5

[11] Levescot A Malamut G Cerf-Bensussan N. Immunopathogenesis and environmental triggers in coeliac disease. Gut. 2022;71:2337–49. 35879049 10.1136/gutjnl-2021-326257 PMC9554150

[12] Sahin Y Sahin DA. The frequency of celiac disease in children with chronic constipation. 2021:383–7.

[13] Guandalini S Assiri A. Celiac disease: a review. JAMA Pediatr. 2014;168:272–8. 24395055 10.1001/jamapediatrics.2013.3858

[14] Caio G Volta U Sapone A. Celiac disease: a comprehensive current review. BMC Med. 2019;17:1–20. 31331324 10.1186/s12916-019-1380-z PMC6647104

[15] Leffler DA Green PHR Fasano A. Extraintestinal manifestations of coeliac disease. Nat Rev Gastroenterol Hepatol. 2015;12:561–71. 26260366 10.1038/nrgastro.2015.131

[16] Dahash BA Alaber OA Sankararaman S. S0129 Association of celiac disease and pancreatitis – a national database study. Am Coll Gastroenterol. 2020;115:S64.

[17] Sadr–Azodi O Sanders DS Murray JA Ludvigsson JF. Patients with celiac disease have an increased risk for pancreatitis. Clin Gastroenterol Hepatol. 2012;10:1136–42.e3. 22801059 10.1016/j.cgh.2012.06.023 PMC3494459

[18] Sanderson E Glymour MM Holmes MV. Mendelian randomization. Nat Rev Methods Primers. 2022;2:6. 37325194 10.1038/s43586-021-00092-5 PMC7614635

[19] Emdin CA Khera AV Kathiresan S. Mendelian randomization. JAMA. 2017;318:1925–6. 29164242 10.1001/jama.2017.17219

[20] Lawlor DA Harbord RM Sterne JAC Timpson N Davey Smith G. Mendelian randomization: using genes as instruments for making causal inferences in epidemiology. Stat Med. 2008;27:1133–63. 17886233 10.1002/sim.3034

[21] Troncone R Discepolo V. Celiac disease and autoimmunity. J Pediatr Gastroenterol Nutr. 2014;59:S9–S11. 24979198 10.1097/01.mpg.0000450394.30780.ea

[22] Lauret E Rodrigo L. Celiac disease and autoimmune‐associated conditions. Biomed Res Int. 2013;2013:127589. 23984314 10.1155/2013/127589 PMC3741914

[23] Sahin Y Evliyaoglu O Erkan T. The frequency of celiac disease in children with autoimmune thyroiditis. Acta Gastroenterol Belg. 2018;81:5–8. 29562371

[24] Şahin Y Şahin S Adrovic A. Serological screening for celiac disease in children with systemic lupus erythematosus. Eur J Rheumatol. 2019;6:142–5. 31070578 10.5152/eurjrheum.2019.18130 PMC6668639

[25] Sahin Y Sahin S Barut K. Serological screening for coeliac disease in patients with juvenile idiopathic arthritis. Arab J Gastroenterol. 2019;20:95–8. 31182344 10.1016/j.ajg.2019.05.005

[26] Sahin Y Cakir MD Isakoca M Aydin Sahin D. Prevalence of celiac disease in children with type 1 diabetes mellitus in the South of Turkey. Iran J Pediatr. 2020;30:e97306.

[27] Beas R Altamirano-Farfan E Izquierdo-Veraza D. Prevalence of celiac disease in systemic lupus erythematosus, Sjogren syndrome and systemic sclerosis: a systematic review and meta-analysis. Dig Liver Dis. 2024;56:1475–82. 38584032 10.1016/j.dld.2024.03.015

[28] Zhao SS Mackie SL Zheng J. Why clinicians should know about Mendelian randomization. Rheumatology (Oxford). 2021;60:1577–9. 33493347 10.1093/rheumatology/keab007

[29] Elsworth B Lyon M Alexander T. The MRC IEU OpenGWAS data infrastructure. BioRxiv. 2020:2020.08. 10.244293.

[30] Kurki MI Karjalainen J Palta P; FinnGen. FinnGen provides genetic insights from a well-phenotyped isolated population. Nature. 2023;613:508–18. 36653562 10.1038/s41586-022-05473-8 PMC9849126

[31] Mbatchou J Barnard L Backman J. Computationally efficient whole-genome regression for quantitative and binary traits. Nat Genet. 2021;53:1097–103. 34017140 10.1038/s41588-021-00870-7

[32] Willer CJ Schmidt EM Sengupta S; Global Lipids Genetics Consortium. Discovery and refinement of loci associated with lipid levels. Nat Genet. 2013;45:1274–83. 24097068 10.1038/ng.2797 PMC3838666

[33] Yan J Zhang Y Zhang X. Increased risk of rheumatoid arthritis in patients with asthma: a genetic association study using two‐sample Mendelian randomization analysis. Arthritis Care Res (Hoboken). 2023;77:178–84. 37465942 10.1002/acr.25193

[34] Bowden J Spiller W Del Greco M F. Improving the visualization, interpretation and analysis of two-sample summary data Mendelian randomization via the radial plot and radial regression. Int J Epidemiol. 2018;47:1264–78. 29961852 10.1093/ije/dyy101 PMC6124632

[35] Hemani G Tilling K Davey Smith G. Orienting the causal relationship between imprecisely measured traits using GWAS summary data. PLoS Genet. 2017;13:e1007081. 29149188 10.1371/journal.pgen.1007081 PMC5711033

[36] Nahler G Bonferroni correction. In Dictionary of Pharmaceutical Medicine. Springer; 2009:18.

[37] Oxentenko AS Rubio-Tapia A. Celiac disease. Mayo Clin Proc. 2019;94:2556–71. 31806106 10.1016/j.mayocp.2019.02.019

[38] Tjon J ML van Bergen J Koning F. Celiac disease: how complicated can it get? Immunogenetics. 2010;62:641–51. 20661732 10.1007/s00251-010-0465-9 PMC2944025

[39] Husby S Koletzko S Korponay‐Szabó IR; ESPGHAN Gastroenterology Committee. European Society for Pediatric Gastroenterology, Hepatology, and Nutrition guidelines for the diagnosis of coeliac disease. J Pediatr Gastroenterol Nutr. 2012;54:136–60. 22197856 10.1097/MPG.0b013e31821a23d0

[40] Therrien A Kelly CP Silvester JA. Celiac disease: extraintestinal manifestations and associated conditions. J Clin Gastroenterol. 2020;54:8–21. 31513026 10.1097/MCG.0000000000001267 PMC6895422

[41] Bultron G Latif U Park A Phatak U Pashankar D Husain SZ. Acute pancreatitis in a child with celiac disease. J Pediatr Gastroenterol Nutr. 2009;49:137–8. 19711503 10.1097/mpg.0b013e318172aad1

[42] Alkhayyat M Saleh MA Abureesh M. The risk of acute and chronic pancreatitis in celiac disease. Dig Dis Sci. 2021;66:2691–9. 32809104 10.1007/s10620-020-06546-2

[43] Ludvigsson JF Montgomery SM Ekbom A. Risk of pancreatitis in 14,000 individuals with celiac disease. Clin Gastroenterol Hepatol. 2007;5:1347–53. 17702659 10.1016/j.cgh.2007.06.002

[44] Balaban DV Enache I Ciochina M Popp A Jinga M. Pancreatic involvement in celiac disease. World J Gastroenterol. 2022;28:2680–8. 35979168 10.3748/wjg.v28.i24.2680 PMC9260863

[45] Brooks SE Golden MH. The exocrine pancreas in kwashiorkor and marasmus. Light and electron microscopy. West Indian Med J. 1992;41:56–60. 1523833

[46] Patel RS Johlin FC Jr Murray JA. Celiac disease and recurrent pancreatitis. Gastrointest Endosc. 1999;50:823–7. 10570344 10.1016/s0016-5107(99)70166-5

[47] Leeds JS Sanders DS. Risk of pancreatitis in patients with celiac disease: is autoimmune pancreatitis a biologically plausible mechanism? Clin Gastroenterol Hepatol. 2008;6:951; author reply 951. 18674737 10.1016/j.cgh.2007.12.025

[48] Balaban DV Mihai A Dima A Popp A Jinga M Jurcut C. Celiac disease and Sjögren’s syndrome: a case report and review of literature. World J Clin Cases. 2020;8:4151–61. 33024773 10.12998/wjcc.v8.i18.4151 PMC7520766

[49] Chang CC Chang YS Wang SH Lin S-Y Chen Y-H Chen JH. Primary Sjogren’s syndrome and the risk of acute pancreatitis: a nationwide cohort study. BMJ Open. 2017;7:e014807. 10.1136/bmjopen-2016-014807 PMC5724124 28801391

