# Supplementary material for: Celiac disease as a risk factor for pancreatitis: Evidence from multivariable Mendelian randomization and mediation analysis
Source: Medicine (Baltimore). 2025 Sep 12;104(37):e44445. doi: 10.1097/MD.0000000000044445 (PMC12440547; doi:10.1097/MD.0000000000044445)

**Supplementary Figure 1 Radial plots to visually depict individual outlier SNPs in the MR analysis**

Colored dots represent outlier SNPs corresponding to different MR methods, while black dots represent non-outlier SNPs.

1. outlier SNPs in the MR analysis exploring the effect of celiac disease on acute pancreatitis in the discovery cohort
2. outlier SNPs in the MR analysis exploring the effect of celiac disease on chronic pancreatitis in the discovery cohort
3. outlier SNPs in the MR analysis exploring the effect of celiac disease on alcohol-induced acute pancreatitis in the discovery cohort
4. outlier SNPs in the MR analysis exploring the effect of celiac disease on alcohol-induced chronic pancreatitis in the discovery cohort
5. outlier SNPs in the MR analysis exploring the effect of celiac disease on acute pancreatitis in the replication cohort
6. outlier SNPs in the MR analysis exploring the effect of celiac disease on chronic pancreatitis in the replication cohort
7. outlier SNPs in the MR analysis exploring the effect of celiac disease on alcohol-induced acute pancreatitis in the replication cohort
8. outlier SNPs in the MR analysis exploring the effect of celiac disease on alcohol-induced chronic pancreatitis in the replication cohort


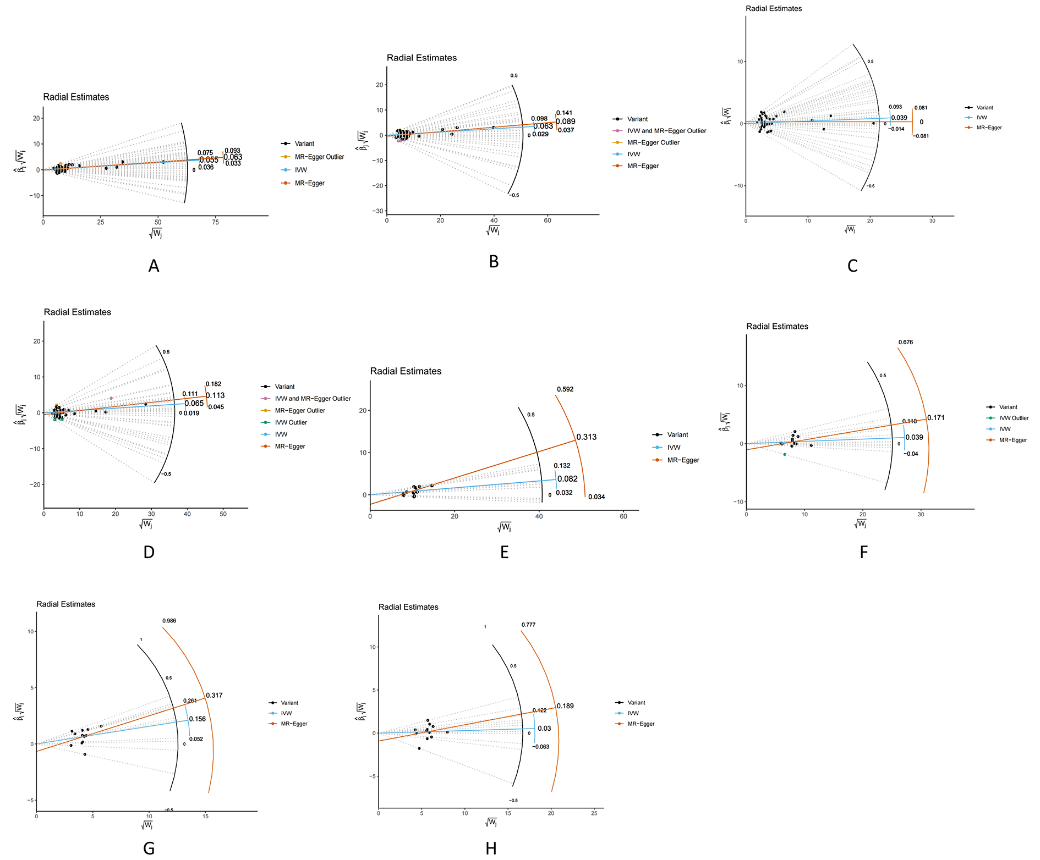


**Supplementary Figure 2 The results of leave-one-out analysis**

A) The results of leave-one-out analysis exploring the effect of celiac disease on acute pancreatitis in the discovery cohort

B) The results of leave-one-out analysis exploring the effect of celiac disease on chronic pancreatitis in the discovery cohort

C) The results of leave-one-out analysis exploring the effect of celiac disease on alcohol-induced acute pancreatitis in the discovery cohort

D) The results of leave-one-out analysis exploring the effect of celiac disease on alcohol-induced chronic pancreatitis in the discovery cohort

E) The results of leave-one-out analysis exploring the effect of celiac disease on acute pancreatitis in the replication cohort

F) The results of leave-one-out analysis exploring the effect of celiac disease on chronic pancreatitis in the replication cohort

G) The results of leave-one-out analysis exploring the effect of celiac disease on alcohol-induced acute pancreatitis in the replication cohort

H) The results of leave-one-out analysis exploring the effect of celiac disease on alcohol-induced chronic pancreatitis in the replication cohort


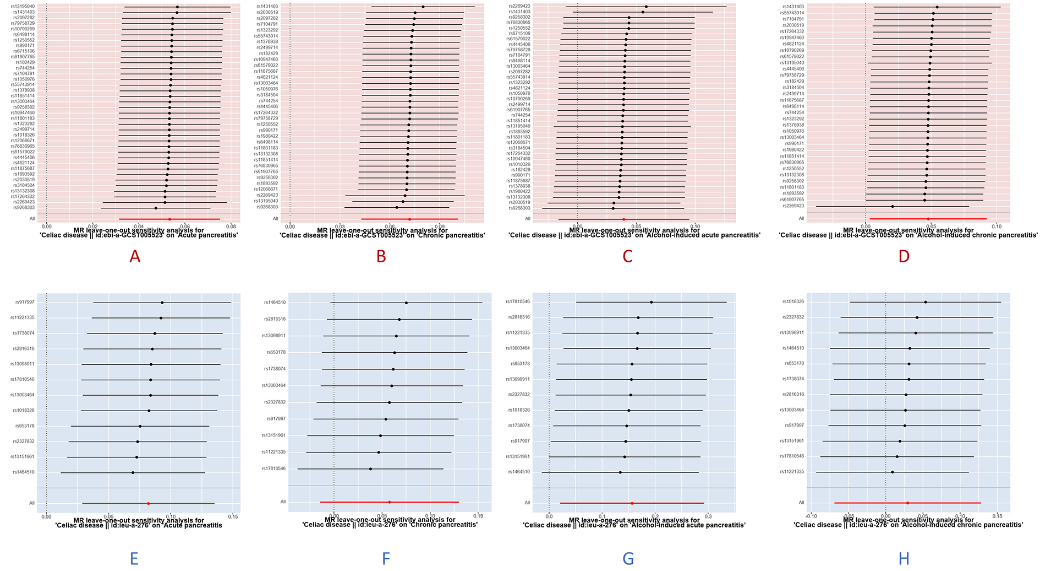

Supplement: Supplementary file 1 [file medi-104-e44445-s001.docx]
